# Assessment of the prognostic and predictive utility of the Breast Cancer Index (BCI): an NCIC CTG MA.14 study

**DOI:** 10.1186/s13058-015-0660-6

**Published:** 2016-01-04

**Authors:** Dennis C. Sgroi, Judy-Anne W. Chapman, T. Badovinac-Crnjevic, Elizabeth Zarella, Shemeica Binns, Yi Zhang, Catherine A. Schnabel, Mark G. Erlander, Kathleen I. Pritchard, Lei Han, Lois E. Shepherd, Paul E. Goss, Michael Pollak

**Affiliations:** Molecular Pathology Unit, Pathology Research Center, Massachusetts General Hospital, MGH East, Molecular Pathology, Research, 149 13th Street, Charlestown, MA 02129 USA; Center for Cancer Research, Massachusetts General Hospital, Charlestown, MA USA; Department of Pathology, Harvard Medical School, Boston, MA USA; NCIC Clinical Trials Group, Queen’s University, Kingston, ON Canada; bioTheranostics, Inc., San Diego, CA USA; Trovagene Inc., San Diego, CA USA; Sunnybrook Odette Cancer Centre and University of Toronto, Toronto, ON Canada; Lady Davis Institute, Jewish General Hospital, McGill University, Montreal, QC Canada

**Keywords:** Breast Cancer Index, HOXB13:IL17BR, Molecular grade index, MA.14

## Abstract

**Background:**

Biomarkers that can be used to accurately assess the residual risk of disease recurrence in women with hormone receptor–positive breast cancer are clinically valuable. We evaluated the prognostic value of the Breast Cancer Index (BCI), a continuous risk index based on a combination of HOXB13:IL17BR and molecular grade index, in women with early breast cancer treated with either tamoxifen alone or tamoxifen plus octreotide in the NCIC MA.14 phase III clinical trial (ClinicalTrials.gov Identifier NCT00002864; registered 1 November 1999).

**Methods:**

Gene expression analysis of BCI by real-time polymerase chain reaction was performed blinded to outcome on RNA extracted from archived formalin-fixed, paraffin-embedded tumor samples of 299 patients with both lymph node–negative (LN−) and lymph node–positive (LN+) disease enrolled in the MA.14 trial. Our primary objective was to determine the prognostic performance of BCI based on relapse-free survival (RFS). MA.14 patients experienced similar RFS on both treatment arms. Association of gene expression data with RFS was evaluated in univariate analysis with a stratified log-rank test statistic, depicted with a Kaplan-Meier plot and an adjusted Cox survivor plot. In the multivariate assessment, we used stratified Cox regression. The prognostic performance of an emerging, optimized linear BCI model was also assessed in a post hoc analysis.

**Results:**

Of 299 samples, 292 were assessed successfully for BCI for 146 patients accrued in each MA.14 treatment arm. BCI risk groups had a significant univariate association with RFS (stratified log-rank *p* = 0.005, unstratified log-rank *p* = 0.007). Adjusted 10-year RFS in BCI low-, intermediate-, and high-risk groups was 87.5 %, 83.9 %, and 74.7 %, respectively. BCI had a significant prognostic effect [hazard ratio (HR) 2.34, 95 % confidence interval (CI) 1.33–4.11; *p* = 0.004], although not a predictive effect, on RFS in stratified multivariate analysis, adjusted for pathological tumor stage (HR 2.22, 95 % CI 1.22–4.07; *p* = 0.01). In the post hoc multivariate analysis, higher linear BCI was associated with shorter RFS (*p* = 0.002).

**Conclusions:**

BCI had a strong prognostic effect on RFS in patients with early-stage breast cancer treated with tamoxifen alone or with tamoxifen and octreotide. BCI was prognostic in both LN− and LN+ patients. This retrospective study is an independent validation of the prognostic performance of BCI in a prospective trial.

**Electronic supplementary material:**

The online version of this article (doi:10.1186/s13058-015-0660-6) contains supplementary material, which is available to authorized users.

## Background

The majority of invasive breast cancers express the estrogen receptor (ER) and/or progesterone receptor (PR), predicting a greater likelihood of response to hormone therapy. However, 25–30 % of early ER+ and/or PR+ breast cancers relapse despite therapy [1]. Classic clinicopathological parameters do not reveal the wide molecular heterogeneity between breast tumors and fail to accurately delineate clinical outcome [2]. Over the past 10 years, gene expression profiling has emerged as a successful clinical strategy to meet the need for better methods to assess the risk of recurrence and to better inform treatment decisions in patients with hormone receptor–positive (HR+) breast cancer [3–12].

The Breast Cancer Index (BCI) is a continuous risk index model of two previously described biomarkers: molecular grade index (MGI) and HOXB13:IL17BR (H:I) ratio [5, 12, 13]. The MGI is a five-gene predictor that recapitulates tumor grade and/or proliferation and is highly prognostic in patients with ER+ breast cancer [5]. H:I, which was developed independent of tumor grade and/or proliferation, is prognostic for early and late distant recurrences and is predictive of adjuvant and extended adjuvant hormonal benefit in patients with early-stage HR+, LN− breast cancer [6, 12, 14] who have received no adjuvant chemotherapy. MGI together with H:I provides more accurate prognosis than either biomarker alone [5]. BCI has been shown to significantly delineate 0- to 10- year risk of recurrence beyond standard clinicopathological factors [9, 12, 13].

The investigators in the NCIC Clinical Trials Group (CTG) MA.14 clinical trial (ClinicalTrials.gov Identifier: NCT00002864) randomized women, regardless of lymph node (LN) status, to tamoxifen (TAM) with or without octreotide LAR [15]. Our aim in this study was to assess the prognostic and predictive value of BCI in women with LN− or LN+ breast cancer who were administered TAM and were enrolled in NCIC CTG MA.14.

## Methods

### Study design

The NCIC CTG MA.14 researchers enrolled 667 postmenopausal women between 1996 and 2000 [15]. Patients were randomly assigned to arm 1 (TAM 20 mg orally once daily for 5 years) or arm 2 [TAM 20 mg orally once daily for 5 years plus octreotide long-acting release (OCT) 90 mg intramuscularly monthly for 5 years (TAM-OCT)]. Patients were stratified by adjuvant chemotherapy (none, concurrent, or sequential), LN status (none, one to three, four or more, or unknown), and receptor status (ER+ and/or PR+, ER− and PR−, or ER and PR unknown). This study was approved by the human research committees of the Massachusetts General Hospital, Queen’s University, and McGill University. All patients provided written informed consent before trial participation. In July 2000, the duration of OCT was reduced from 5 to 2 years because of a greater incidence of gallbladder toxicity in the OCT arm of National Surgical Adjuvant Breast and Bowel Project B-29. The conduct of the study was overseen by an NCIC CTG study team; Novartis Canada, which provided the OCT; and the independent NCIC CTG Data and Safety Monitoring Committee.

### Study population

Patients had histologically proven adenocarcinoma of the breast with satisfactory surgical removal of the tumor by lumpectomy or total mastectomy [15]. Patients were to have no previous or concurrent malignancies except adequately treated carcinoma of the skin (basal cell), cervix, endometrium, colon, or thyroid treated more than 5 years before study entry, and they had to have a life expectancy of at least 5 years. Tumors could be ER+ and/or PR+ (biochemical value ≥10 fmol/mg or positive by immunohistochemistry), negative, or unknown. Baseline serum was assessed for insulin-like growth factor 1, insulin-like growth factor-binding protein 3, and C-peptide for 646 patients (96.9 %), and 25-hydroxy vitamin D was centrally assessed for 607 of the MA.14 patients (91 %) [15, 16].

### Study endpoints

The primary endpoint of the MA.14 trial was event-free survival (EFS). Events included recurrence of disease, second malignancy, or death due to any cause. Overall survival (OS) was a secondary endpoint. Relapse-free survival (RFS) was also a secondary endpoint of the MA.14 trial, and it was defined as the time from randomization to the time of recurrence of the primary disease alone, including local and ipsilateral nodal recurrence, excluding contralateral breast cancer, with censoring at longest follow-up or death due to another cause. RFS is the primary endpoint for this investigation.

### MA.14 trial experience

EFS was the primary endpoint of MA.14. At the final analysis at a median of 7.9 years, the stratified hazard ratio (HR) for TAM + OCT to TAM was 0.93 [95 % confidence interval (CI) 0.71–1.22; *p* = 0.62] [15]. OS had an HR of 0.97 (95 % CI 0.69–1.37; *p* = 0.86). The RFS HR was 0.84 (95 % CI, 0.59–1.18; *p* = 0.31). Patients allocated to octreotide had an absolute 2.7 % lower rate of recurrence during the study period. At the median 9.8-year trial follow-up, the RFS HR was 0.87 (95 % CI 0.63–1.21; *p* = 0.40). The median 9.8-year trial follow-up was used for these investigations.

### Study objectives

The primary objective of this investigation was to examine whether BCI has a prognostic association with RFS. We used pooled data across both MA.14 treatment arms. Secondary objectives included exploration of whether the BCI classification (low vs. medium and/or high) had a predictive effect on RFS.

### BCI analytic methods

For each formalin-fixed, paraffin-embedded tumor sample, three 8-μm tissue sections were subjected to gross macrodissection to enrich for tumor content. RNA extraction, amplification, and real-time time quantitative polymerase chain reaction (RT-qPCR) were performed at bioTheranostics Inc. (San Diego, CA, USA), a Clinical Laboratory Improvements Amendments–certified laboratory, with researchers blinded to clinical outcome [5, 6, 17]. The prespecified BCI genes, primer and probe sequences, RT-qPCR, and calculation of H:I and MGI were performed as previously described [5, 6]. Patient samples were excluded if there was insufficient RNA. The average cycle threshold for normalizing genes was >28.5. A continuous risk model called BCI was previously built by combining H:I and MGI. BCI was categorized into three levels: low-risk BCI <5, intermediate risk 5 ≤ BCI < 6.4, and high-risk BCI ≥6.4 [13]. These prespecified BCI cutoffs were chosen on the basis of previously established and validated cutoffs for LN−, HR+ patients with breast cancer who did not receive adjuvant chemotherapy [5, 13]. During this study, a second, “optimized” linear model of BCI (linear BCI) was developed and a post hoc analysis of the prognostic performance of linear BCI was performed using the specified risk groups as described previously [9, 12].

### Statistical analyses

We used Fisher’s exact test to examine whether there were significant imbalances by treatment arm and stratification factors in who was or was not assessable for BCI. A histogram of continuous gene expressions for BCI, H:I, and MGI was created to examine whether a Box-Cox transformation should be considered to reduce asymmetry and stabilize variances. In univariate testing, we used a stratified log-rank test statistic. Graphical depiction was created with an unadjusted Kaplan-Meier plot and adjusted Cox survivor plot, where adjustment was by MA.14 trial treatment, stratification factors, and other factors with significant multivariate associations with RFS. Exploratory, stratified, stepwise forward Cox regressions were performed, whereby baseline patient and tumor characteristics were added if two-sided *p* ≤ 0.05 with a likelihood ratio criterion test statistic has approximately a chi-square distribution with 1 degree of freedom. The Cox assumption of proportional hazards was examined with plots of log-cumulative hazards versus follow-up time. Factors with significant time-to-event RFS associations and subgroups with crossing of cumulative hazards plots would have been considered to have substantive nonproportionality. An adjusted Cox model was used to estimate adjusted 5- and 10-year RFS [18, 19].

## Results

Of the 667 MA.14 patients, 299 patients had banked tumor blocks. The patients with banked tumor blocks were not significantly different from those without, when classified by treatment arm and stratification factors; by LN status (*p* = 0.90); hormone receptor status (*p* = 0.19); or adjuvant chemotherapy (*p* = 0.90). The patients had similar median follow-up by trial arm of 10.01 years on TAM and 10.12 years on TAM-OCT, compared with the median follow-up of 9.8 years in the full trial. The patient group of LN−, HR+ patients who did not receive adjuvant chemotherapy, the type of patients from whom BCI was initially developed, was similar across treatment arms, with 63 patients on TAM and 58 patients on TAM-OCT.

From the 299 patients with blocks, 292 samples passed internal analytical quality control (REporting recommendations for tumor MARKer prognostic studies [REMARK] diagram in Additional file 1: Figure S1). The RT-qPCR histograms for BCI (Additional file 2: Figure S2a), H:I (Additional file 2: Figure S2b), and MGI (Additional file 2: Figure S2c) indicated reasonably symmetrical distributions. Baseline patient characteristics are provided in Table 1. Each trial arm had 146 patients assessed, and the patients were similar by trial arm. Of note, 51 % of the investigative group were LN−, 92 % were HR+, and 35 % received adjuvant chemotherapy.

**Table 1:** Baseline patient and tumor characteristics

	Tamoxifen-treated (*n* = 146)		Tamoxifen + octreotide–treated (*n* = 146)		Total (*n* = 292)	
	Number	%	Number	%	Number	%
Age, yr						
<60	71	49	65	45	136	47
≥60	75	51	81	55	156	53
Race						
Caucasian	142	97	137	94	279	96
Non-Caucasian	4	3	9	6	13	4
Performance status						
0, unknown	106	73	118	81	224	77
1, 2	40	27	28	19	68	23
Tumor pathologic classification						
0, 1, in situ	87	60	91	62	178	61
2, 3A, 4, unknown	59	40	55	38	114	39
Node pathology classification						
0	74	51	75	51	149	51
1, 2, unknown	72	49	71	49	143	49
Breast surgery type						
Total mastectomy	50	34	58	40	108	37
Other, segmental mastectomy	96	66	88	60	184	63
Number of positive axillary nodes (R)						
0	75	51	74	51	149	51
1–3, 4+, unknown	71	49	72	49	143	49
Estrogen/progesterone receptor status (R)						
Negative, unknown	14	10	10	7	24	8
Positive	132	90	136	93	268	92
Adjuvant chemotherapy (R)						
None	96	66	94	64	190	65
Concurrent, sequential	50	34	52	36	102	35

**Fig. 1:**
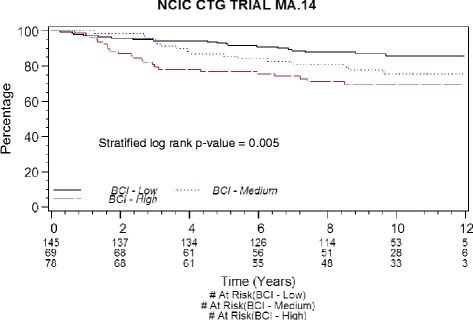
Risk-free survival Kaplan-Meier plot of Breast Cancer Index (BCI). *CTG* Clinical Trials Group

BCI risk groups had a significant univariate association with RFS (stratified log-rank *p* = 0.005; unstratified log-rank *p* = 0.007) (Fig. 1), with the expected direction that low BCI had the highest RFS, medium BCI had intermediate RFS, and high BCI had lowest RFS. In stratified multivariate analysis, larger pathologic tumor stage (HR 2.22, 95 % CI 1.22–4.07; *p* = 0.01), and higher continuous BCI (HR 2.34, 95 % CI 1.33–4.11; *p* = 0.004) were associated with worse RFS (Table 2). The interaction of BCI and trial treatment was not significant (*p* = 0.28). The interactions of treatment with LN status (*p* = 0.88) and with adjuvant chemotherapy (*p* = 0.81) also were not significant. There was no evidence of substantive nonproportional hazards.

**Table 2:** Stratified Cox stepwise multivariate model for effects of factors on RFS

Factor	Hazard ratio (95 % CI)	*p* Value^a^
Pathologic T status T2 or higher vs. lower than T2	2.22 (1.22–4.07)	0.01
Continuous cubic BCI	2.34 (1.33–4.11)	0.004

*T* tumor, *BCI* Breast Cancer Index, *CI* confidence interval

^a^ *p* Value is based on the likelihood ratio criterion likelihood ratio criterion test statistic has approximately a chi-square distribution with 1 degree of freedom.

**Fig. 2:**
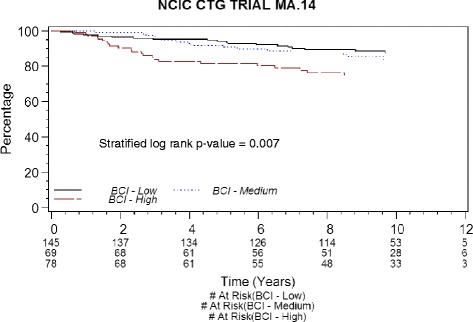
Risk-free survival adjusted Cox survivor plot by Breast Cancer Index (BCI), adjustments by treatment, MA.14 stratification factors, and pathologic tumor stage. *CTG* Clinical Trials Group

The adjusted Cox survivor plot (Fig. 2) depicts categorical BCI experience (*p* = 0.007) adjusted for the effects of treatment, stratification factors (ER and PR status, LN status, and adjuvant chemotherapy), and pathologic tumor stage. The HR of intermediate BCI to low BCI was 1.28 (95 % CI 0.65–2.52), while that of high BCI to low BCI was 2.53 (95 % CI 1.36–4.71). The BCI low-risk group had 5- and 10-year RFS rates of 94 % and 87.5 %, the BCI intermediate-risk group had 5- and 10-year RFS rates of 91.8 % and 83.9 %, and the BCI high-risk group had 5- and 10-year RFS rates of 81.5 % and 74.7 % (Table 3).

**Table 3:** Adjusted Cox estimates of 5- and 10-year relapse-free survival by BCI categories

BCI risk	Number of patients (%)	5-year RFS^a^	10-year RFS^a^
Low	145 (50)	94 %	87.5 %
Intermediate	69 (24)	91.8 %	83.9 %
High	78 (27)	81.5 %	74.7 %

*BCI* Breast Cancer Index, *RFS* risk-free survival

^a^Adjusted Cox estimates of RFS with adjustment for treatment, MA.14 stratification factors, and pathologic tumor stage

At the time of this study, an emerging optimized linear model of BCI (linear BCI) was developed and validated in several independent cohorts [9, 12]. A post hoc analysis with 116 LN+ patients who had HR+ tumors indicated that higher continuous linear BCI was associated with shorter RFS (*p* = 0.002. The results of this univariate analysis are depicted in Additional file 3: Figure S3.

## Discussion

We previously showed that BCI predicts risk of recurrence in patients with LN−, ER+ breast cancer [9, 12, 13]. In this retrospective analysis of a nested MA.14 study, BCI had a strong prognostic association with disease recurrence in postmenopausal patients with LN− and LN+ breast cancer treated with TAM. The probability of being disease-free at 10 years was 87.5 % for low BCI, 83.9 % for intermediate BCI, and 74.7 % for high BCI. Our findings are consistent with those described in previous reports [9, 13, 20]. In the Stockholm trial, the 10-year distant metastasis rates in TAM–treated, ER+, LN− patients were 1.7 %, 16.9 %, and 20 % for low, intermediate, and high BCI, respectively [13]. In another large case–control study, 10-year risk of breast cancer death among patients with ER+, LN−, breast cancer treated with TAM were 3.5 % , 7.0 %, and 12.9 % for low, intermediate, and high BCI, respectively [20]. The RFS rates for BCI in our study are lower than in previous trials [9, 13]; however, they are adjusted for the effects of potential confounders between studies (trial treatment, LN status, adjuvant chemotherapy, and hormone receptor status). As BCI was developed in ER+ patients, the inclusion of a substantial (8 %) number of ER−/ER unknown patients in the present study likely impacted the prognostic performance of BCI. Furthermore, this difference in performance is likely attributable to the inclusion of the higher-risk LN+ patients in the present study cohort as compared with previous cohorts.

Patients with axillary node metastases are traditionally considered as having a poor prognosis [1]. Interestingly, half of the patients in our trial were classified as low risk, again confirming that even among LN+ patients there is a subgroup of patients with a good prognosis [21–24]. Because most patients with LN+ breast cancer in our study received adjuvant chemotherapy or hormone therapy, we could not evaluate the prognostic value of BCI in patients with untreated LN+ disease. A recent subgroup analysis of the LN+ MA.14 patients revealed that BCI was a significant prognostic factor for HR+ patients who were treated with TAM [25]. These findings could have important implications for treatment decisions because not all patients with LN+ breast cancer require aggressive treatment [21, 24]. However, validation of these findings in additional cohorts of patients with LN+ breast cancer is warranted.

At the time of this study, an emerging optimized linear model of BCI (linear BCI) was developed and validated in several independent cohorts [9, 13]. Our post hoc analysis of linear BCI in the MA.14 cohort revealed that linear BCI had prognostic results similar to those reported in other cohorts [9, 12]. The MA.14 trial precluded robust comparisons with the unoptimized BCI.

Our study has limitations. With the two trial arms having similar RFS experience, it would have been more difficult to see a predictive effect for BCI. All MA.14 patients were postmenopausal, so our results are not generalizable to premenopausal women. Around one-third of women in our trial received adjuvant chemotherapy in addition to TAM, and the multivariate analysis was stratified by receipt of chemotherapy; however, the number of patients in the chemotherapy subgroup is too small to infer what effect, if any, chemotherapy had on disease outcomes. As well, the number of patients available for analysis in our study was relatively small and did not allow us to accurately quantify the differences among LN− and LN+ patients or, more specifically, the experience in our original target population (ER+, LN−, no chemotherapy subgroup) because the latter group of patients had only 11 RFS events, which is too few to allow further subdivision by BCI risk group classification. Our exploratory analysis showed that BCI continued to have a significant prognostic effect in TAM-treated patients after stratification or adjustment by standard factors, including LN status and tumor size. Our retrospective analysis included only a subset of patients from the MA.14 trial, although we found that those assessed for BCI were similar to those who were not. Thus, the prognostic and predictive effects of BCI might differ because of the inclusion of disease-free survival events such as locoregional breast recurrences. Further research is needed to show if these results can be extended to other populations of breast cancer patients, particularly younger women.

In summary, for TAM-treated patients, BCI has been shown to have a prognostic but not predictive effect on RFS that extends beyond traditional breast cancer parameters such as LN status, tumor size, and treatment. The results of this study affirm those published recently by our group and others and will contribute to the assessment of the clinical utility of BCI evaluation in guiding adjuvant therapy choices for patients.

## Conclusions

BCI had a strong prognostic effect on RFS in patients with early-stage breast cancer treated with tamoxifen alone or with tamoxifen and octreotide. BCI was prognostic in both LN− and LN+ patients. This retrospective study is an independent validation of the prognostic performance of BCI in a prospective trial.

## Additional files

Additional file 1: Figure S1. REMARK diagram for BCI investigations. (PDF 51 kb) Additional file 2: Figure S2.
**a** Frequency histogram of BCI. **b** Frequency histogram of H:I. **c** Frequency histogram of MGI. (ZIP 117 kb) Additional file 3: Figure S3. RFS Kaplan-Meier plot of linear BCI for lymph node–positive patients. (PDF 90 kb)

## Abbreviations

BCI: Breast Cancer Index
CTG: Clinical Trials Group
EFS: event-free survival
ER: estrogen receptor
H:I: HOXB13:IL17BR
HR: hazard ratio
HR+: hormone receptor–positive
LN: lymph node
MGI: molecular grade index
OCT: octreotide long-acting release
PR: progesterone receptor
REMARK: REporting recommendations for tumor MARKer prognostic studies
RFS: relapse-free survival
RT-qPCR: real-time quantitative polymerase chain reaction
TAM: tamoxifen
