# Supplementary material for: Assessment of the prognostic and predictive utility of the Breast Cancer Index (BCI): an NCIC CTG MA.14 study
Source: Breast Cancer Res. 2016 Jan 4;18:1. doi: 10.1186/s13058-015-0660-6 (PMC4700696; doi:10.1186/s13058-015-0660-6)
Supplement: Additional file 1: Figure S1. — REMARK diagram for BCI investigations. (PDF 51 kb) [file 13058_2015_660_MOESM1_ESM.pdf]

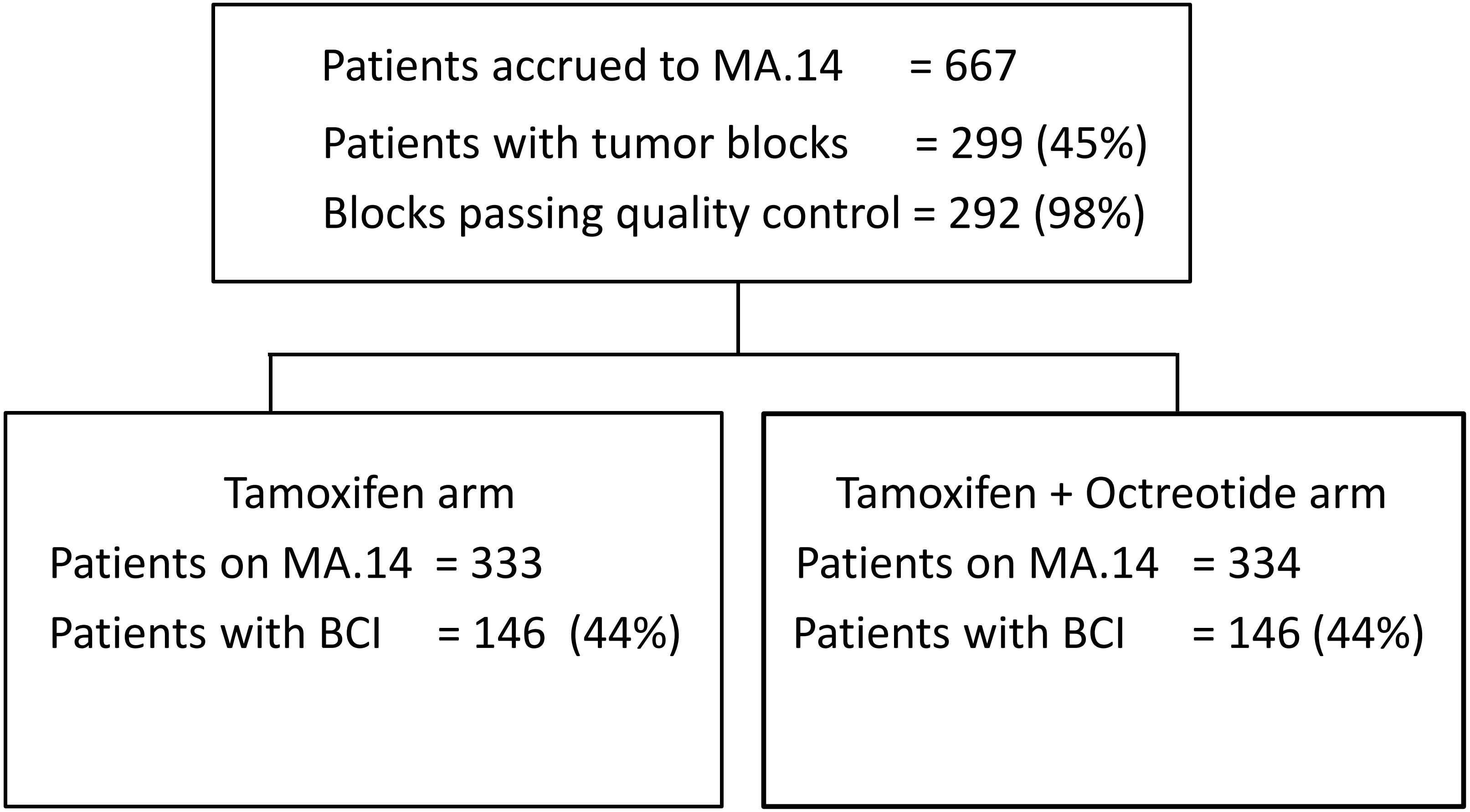
Patients accrued to MA.14 = 667
Patients with tumor blocks = 299 (45%)
Blocks passing quality control = 292 (98%)
Tamoxifen arm
Patients on MA.14 = 333
Patients with BCI = 146 (44%)
Tamoxifen + Octreotide arm
Patients on MA.14 = 334
Patients with BCI = 146 (44%)
