# Supplementary figures and images for: Assessment of the prognostic and predictive utility of the Breast Cancer Index (BCI): an NCIC CTG MA.14 study
Source: Breast Cancer Res. 2016 Jan 4;18:1. doi: 10.1186/s13058-015-0660-6 (PMC4700696; doi:10.1186/s13058-015-0660-6)

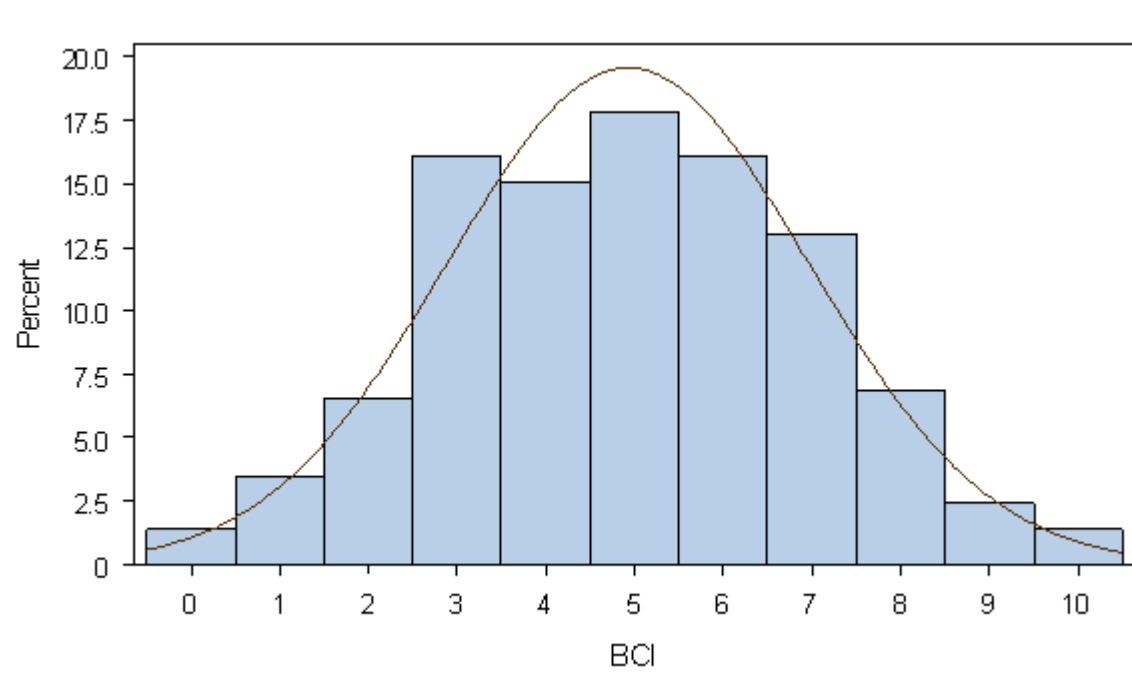

Supplement: Additional file 2: Figure S2. — a Frequency histogram of BCI. b Frequency histogram of H:I. c Frequency histogram of MGI. (ZIP 117 kb) [file 13058_2015_660_MOESM2_ESM.zip › Supplemental-Figure_2a 8-31-15.pdf]

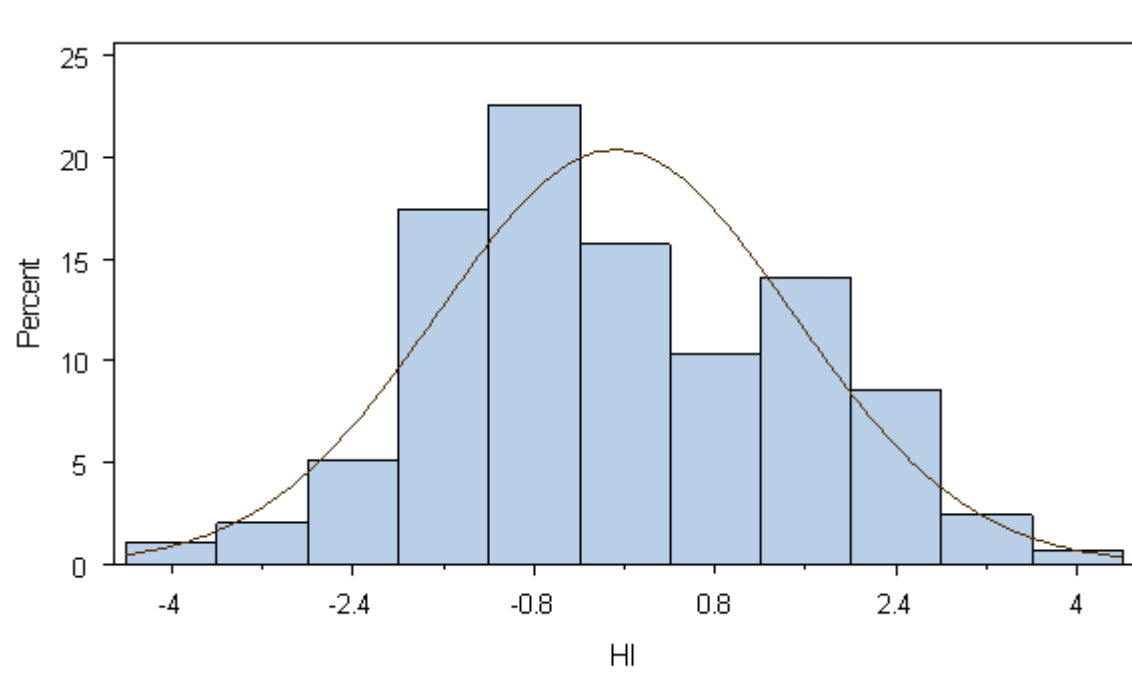

Supplement: Additional file 2: Figure S2. — a Frequency histogram of BCI. b Frequency histogram of H:I. c Frequency histogram of MGI. (ZIP 117 kb) [file 13058_2015_660_MOESM2_ESM.zip › Supplemental-Figure_2b 8-31-15.pdf]

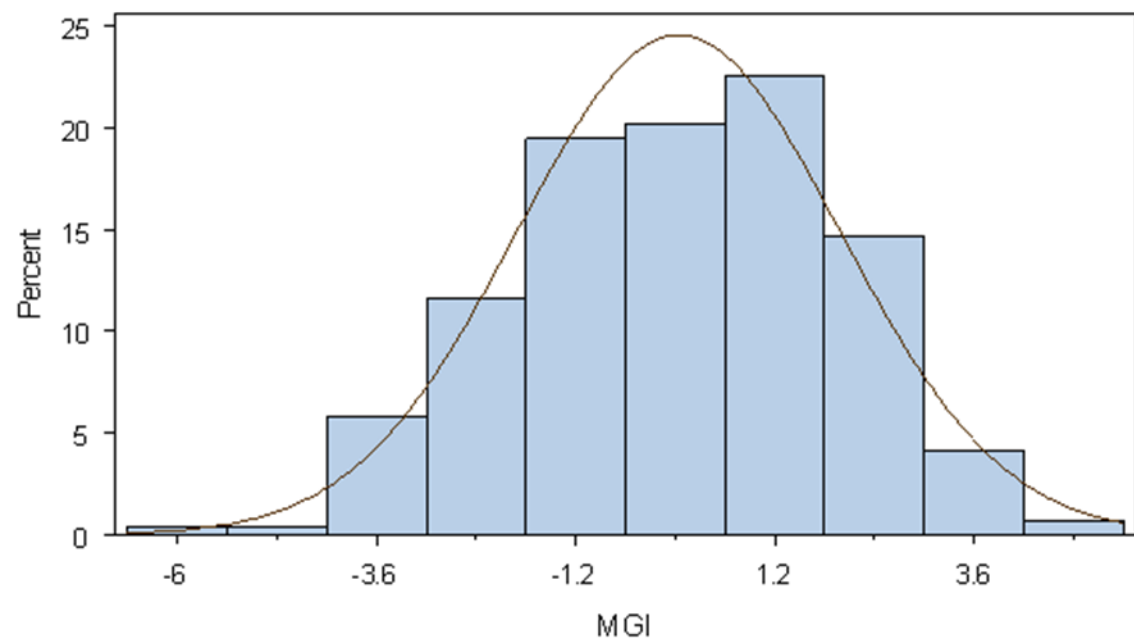

Supplement: Additional file 2: Figure S2. — a Frequency histogram of BCI. b Frequency histogram of H:I. c Frequency histogram of MGI. (ZIP 117 kb) [file 13058_2015_660_MOESM2_ESM.zip › Supplemental-Figure_2c 8-31-15.pdf]

Kaplan-Meier Plot for RFS by Categorical Linear-BCI

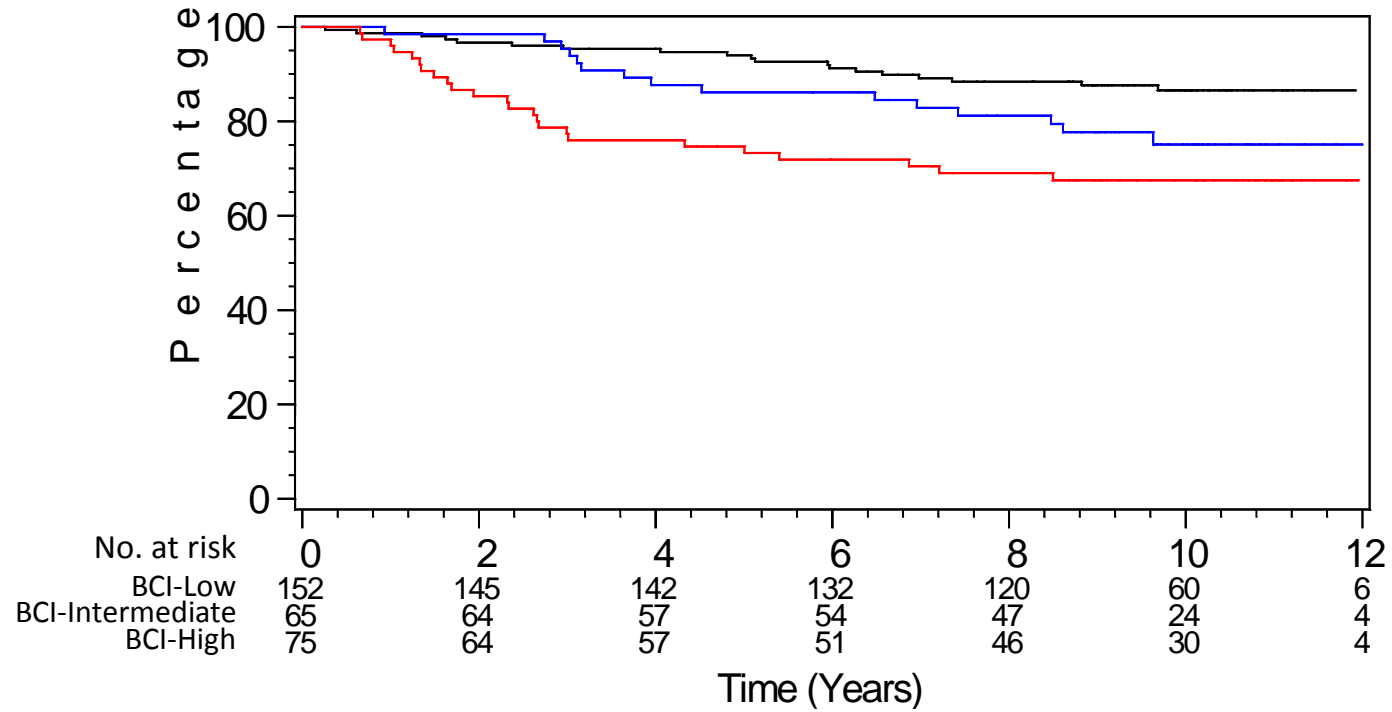

Supplement: Additional file 3: Figure S3. — RFS Kaplan-Meier plot of linear BCI for lymph node–positive patients. (PDF 90 kb) [file 13058_2015_660_MOESM3_ESM.pdf]
